# IsoWeb: A Bayesian Isotope Mixing Model for Diet Analysis of the Whole Food Web

**DOI:** 10.1371/journal.pone.0041057

**Published:** 2012-07-27

**Authors:** Taku Kadoya, Yutaka Osada, Gaku Takimoto

**Affiliations:** 1 Center for Environmental Biology and Ecosystem Studies, National Institute for Environmental Studies, Tsukuba-City, Ibaraki, Japan; 2 Department of Ecosystem studies, School of Agriculture and Life Science, University of Tokyo, Bunkyo-ku, Tokyo, Japan; 3 Department of Biology, Faculty of Science, Toho University, Funabashi, Chiba, Japan; National Institute of Water & Atmospheric Research, New Zealand

## Abstract

Quantitative description of food webs provides fundamental information for the understanding of population, community, and ecosystem dynamics. Recently, stable isotope mixing models have been widely used to quantify dietary proportions of different food resources to a focal consumer. Here we propose a novel mixing model (IsoWeb) that estimates diet proportions of all consumers in a food web based on stable isotope information. IsoWeb requires a topological description of a food web, and stable isotope signatures of all consumers and resources in the web. A merit of IsoWeb is that it takes into account variation in trophic enrichment factors among different consumer-resource links. Sensitivity analysis using realistic hypothetical food webs suggests that IsoWeb is applicable to a wide variety of food webs differing in the number of species, connectance, sample size, and data variability. Sensitivity analysis based on real topological webs showed that IsoWeb can allow for a certain level of topological uncertainty in target food webs, including erroneously assuming false links, omission of existent links and species, and trophic aggregation into trophospecies. Moreover, using an illustrative application to a real food web, we demonstrated that IsoWeb can compare the plausibility of different candidate topologies for a focal web. These results suggest that IsoWeb provides a powerful tool to analyze food-web structure from stable isotope data. We provide R and BUGS codes to aid efficient applications of IsoWeb.

## Introduction

Description of food webs provides fundamental information about ecological systems. Trophic interactions in ecological communities affect species persistence, dynamics and stability of communities [1], [2], and material flows through ecosystems [3], [4]. To describe a food web, either qualitative or quantitative approaches have been used [5]. The topological web is a qualitative description of food webs, consisting of a set of binary (i.e., presence or absence) trophic links among species or trophic guilds in an ecological community. The energy flow web and the functional web are the quantitative description of food webs. The energy flow web describes the importance of each trophic link in a topological web with respect to the amount of energy that flows through the link. The functional web depicts those species and trophic links that strongly influence the dynamics and structure of communities, such as trophic cascades. Although quantitative descriptions of food webs are more preferable for inferring the functioning of food webs [6], [7], it is notoriously laborious to obtain a quantitative description of a food web.

As a step toward obtaining a quantitative food web, here we propose a novel method for estimating diet proportions of all consumers in a food web by utilizing stable isotope information. Recent technological advances have greatly increased applications of stable isotopes to ecological studies, including determining consumer's diet (e.g., [8]), estimating food-chain length [9], and evaluating material flux through ecosystems [10]. It is becoming possible to obtain stable isotope signatures of most species in a food web (e.g., [11]). This suggests a possibility to estimate dietary proportions of all consumers in a food web by integrating stable isotope information of all consumers and resources in the web. The method that we propose expands on previous Bayesian isotope mixing models designed to estimate dietary proportions of a particular consumer (MixSIR [12], SIAR [13]). Unlike previous models, our Bayesian mixing model (hereafter IsoWeb) exploits stable isotope information of all consumers and resources in a food web to estimate dietary proportions of every consumer in the web. This approach will not only allow the simultaneous estimation of dietary proportions of all consumers in a food web, but may also enable more accurate and precise estimates by taking advantage of a large quantity of stable isotope data from a food web.

To develop and demonstrate IsoWeb, our objectives in this paper are threefold. First, we describe the model structure of IsoWeb. Second, we evaluate the performance of IsoWeb (i.e., the accuracy and precision of IsoWeb's estimates) by conducting sensitivity analysis against variation in food-web parameters and sample size and against the topological uncertainty of food webs. For sensitivity analysis against variation in food-web parameters and sample size, we generate a large number of test data sets from many hypothetical food webs assuming realistic values of food–web parameters, such as the number of species and connectance. Determining topological food webs in a field likely involves uncertainty, such as wrong assumption of false links, and omission of existent links or species. In addition, due to difficulties in detailed taxonomic identification, it is usual to aggregate similar species and taxa into a trophospecies that represents a single node of a topological web. To evaluate sensitivity against topological uncertainty, we use data from real food webs, which we modify to incorporate topological uncertainty including the assumption of wrong (non-existent) links, the omission of existent links or species, and species aggregation into trophospecies. Third, we demonstrate an application of IsoWeb to a real food web with real stable isotope data. As an example, we use a case study of a Kenyan wooded grassland food web [11], from which its topological web and stable isotope information of constituent consumers and resources are available. Using this example, we (1) compare the estimates of IsoWeb with the original estimates of dietary proportions in the primary literature [11] (calculated with a classical isotope mixing model [11]) and (2) demonstrate the potential of IsoWeb in evaluating alternative food–web topologies.

## Methods

### Model Structure

We consider a general situation in which consumer *i* of a target food web utilizes *M_i_* resources, and *N* measurements of stable isotope ratios of *J* elements are taken from each consumer and each resource:

*X_ij_*  =  observed isotope ratio of element *j* in consumer *i*; normally distributed with mean *s_ij_* and variance *σ_ij_^2^*.

*s_ij_*  =  mean of isotope ratio of element *j* in consumer *i*.

*σ_ij_^2^* =  residual variances of isotope ratio of element *j* in consumer *i*.


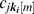
  =  trophic enrichment factor of element *j* for the trophic link from resource ***k*** *_i_*[*m*] to consumer *i* (***k*** *_i_*[*m*] is the *m*th resources of consumer *i* ).


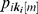
  =  dietary proportion of resource ***k*** *_i_*[*m*] of consumer *i*.


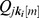
  =  observed concentration of element *j* in resource ***k*** *_i_*[*m*].

The mixing model is formulated as follows:


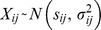



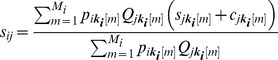



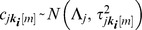



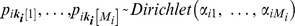


where 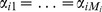 are the parameters of a Dirichlet prior (i.e., a multivariate generalization of the Beta distribution), and *Λ_j_* and 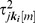 is the mean and variance of prior distribution to which trophic enrichment factors of element *j* across all trophic links included in food web are assumed to follow. Trophic enrichment factor is known to vary substantially across links and food webs but to approximately follow to normal distribution [9]. We used upper and lower cases here to represent data and parameters, respectively.

The model was fitted using the Bayesian simulation-based method (Markov-chain Monte Carlo, MCMC) in WinBUGS 1.4 [14] in combination with R release 2.13.1 (package R2WinBUGS). We supply R and BUGS codes online (Supplement S1). Adopting a Bayesian approach, IsoWeb can incorporate external (prior) information, if any, about parameters to reduce uncertainty in parameter estimation.

### Priors and MCMC Simulations

We assigned vague priors for the parameters to be estimated. We used normal priors for the mean of isotope ratios and gamma priors for the residual variances, and the Dirichlet distribution with noninformative parameters for dietary proportions. We assumed that the trophic enrichment factors of carbon and nitrogen followed a normal distribution with mean 0.8 for carbon, and that with mean 3.4 for nitrogen. The standard deviation of these normal distributions (i.e., 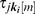) are assumed to follow a half-Cauchy distribution of a large scale parameter value, 25 [15], for both carbon and nitrogen. As for the priors of dietary proportions, we used a Dirichlet distribution with parameters 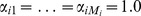, which correspond to noninformative uniform priors. The numbers of MCMC steps were 10000, burn–ins 5000, and thin numbers 5. Convergence was assessed by whether the R–hat indicator of each parameter had reached a value near 1 [16].

To check whether different prior distributions for trophic enrichment factors might affect IsoWeb's estimates, we compared performance between IsoWeb assuming normal and uniform priors, by conducting sensitivity analysis against food-web parameters and sample size (see below for sensitivity analysis). We assigned uniform distributions of [−2.2, 3.0] and [1.4 5.4] as priors for carbon and nitrogen, respectively (the ranges were chosen as mean ±2SD, with mean and SD values taken from literature [9]). We found that IsoWeb with uniform priors performed slightly worse in accuracy but equally well in precision, compared to IsoWeb with normal priors (Fig. S1). In addition, patterns of sensitivities to food-web parameters and sample size were similar between IsoWeb with uniform and normal priors (Fig. S2). Thus we show only the results of IsoWeb with normal priors in the following analyses.

### Evaluation of Model Performance

We conducted sensitivity analysis against variation in food-web parameters and sample size. We generated hypothetical topological webs using the niche model [17]. The niche model produces realistic food-web topology from two food-web parameters: the number of species *S*, and connectance *C*. Although IsoWeb is designed to accommodate food web topologies with cannibal links, we removed cannibal links from the webs generated with the niche model to simplify the generation of virtual stable-isotope data (Supplement S2). To generate realistic test data sets, we chose the following ranges for *S*, *C*, number of samples (*n*), and residual variances for carbon and nitrogen (*σ_ij_* ^2^): *S* = 10–30, *C* = 0.05–3, *n* = 5–50, and *σ_ij_* ^2^ = 0.1–10.0 (for carbon and nitrogen). Assuming parameter values randomly drawn from these ranges, a total of 1500 hypothetical food webs and associated test data sets were generated. We applied IsoWeb to each of 1500 test data sets, and estimated dietary proportions. For each data set, estimated dietary proportions were linearly regressed onto their true values. We used the slope coefficient of this regression as an index of estimation accuracy, and the R-squared as an index of precision.

We conducted the second sensitivity analyses of IsoWeb against the topological uncertainty of food webs. We used topological data from the Benguela food web (29 species and 203 links [18]), the Coachella Valley food web (26 species and 228 links [19]) and the Small Reef food web (50 species and 556 links [20]). The Benguela food web and Small Reef food web have dietary proportions of each species, whereas the Coachella Valley food web does not. We therefore generated hypothetical dietary proportions for the Coachella Valley food web (as we did to generate hypothetical food web data, see below). These dietary proportions of these food webs were treated as true values, to which estimates from IsoWeb were compared. Neither food webs have isotope information. We thus generated virtual data of carbon and nitrogen stable isotope ratios (Supplement S2). Put simply, we first assigned isotope ratios for all basal species by drawing random values from uniform distributions with realistic ranges of isotope ratios (−30 to 0 for carbon, 0 to 5 for nitrogen). We then sequentially calculated isotope ratios for consumer species at higher trophic levels from isotope ratios of their resources according to the true dietary proportions. Assuming the residual variances equal to one for carbon and nitrogen, ten samples of isotope-ratio data were generated for each species. Next we introduced topological uncertainty into this data set. We separately addressed four types of topological uncertainty: (1) false assumption of non-existent links, (2) omission of existent links, (3) omission of existent species, and (4) aggregation of species into trophospecies. We generated test data sets with various levels of topological uncertainty, by changing the number of false links, omitted links, or omitted species within 20% of the number of existent links or species. Thus the number of false or omitted links in the test data sets ranged from one to 41 for the Benguela food web, one to 46 for the Coachella Valley web, and one to 111 for the Small Reef web. The number of omitted species ranged from one to six for the Benguela web, one to five for the Coachella Valley web, and one to 10 for the Small Reef web. Ten random combinations of false links, omitted links, or omitted species were chosen at every level of topological uncertainty (total 880, 970 and 2,320 data sets for the Benguela, the Coachella Valley and the Small Reef food webs, respectively). We assumed such false links that were likely by nitrogen stable isotope ratios (i.e., a link from a consumer to a resource whose mean nitrogen isotope ratio was lower than consumer's). For trophic aggregation, we employed the aggregation method described in Yodzis and Winemiller [21]. This aggregation method was based on trophic similarity between trophic nodes (species or trophospecies). The similarity was calculated using the Jaccard index. Trophic links from a species as a resource and those as a consumer were treated additively (additive topological similarity [21]). Trophospecies were defined as clusters identified from a cluster analysis in which the similarity between clusters is the average similarity over all pairs of every original species in each cluster (ASBC cluster similarity [21]). To generate test data sets with various levels of trophic aggregation, we decreased the threshold for aggregation from 1 (no aggregation) to 0.5 (aggregation of species and clusters within 0.5 similarity), and gained one data set every time a new aggregation occurred (total 19, 18 and 23 data sets for the Benguela, the Coachella Valley and the Small Reef food webs, respectively). When species or clusters were aggregated, their isotope data were also aggregated. We calculated the true dietary proportions of aggregated webs from those of the original un-aggregated web. We calculated the true dietary proportions of aggregated webs only for un-aggregated consumers, because we could not calculate the dietary proportions of an aggregated consumer without knowing the relative contributions of material flows into individual consumers that made up the aggregated consumer. We then applied IsoWeb to each of these test data sets and evaluated the performance of IsoWeb by comparing the IsoWeb's estimates of dietary proportions with the true dietary proportions. For the estimates from each data set, estimated dietary proportions were linearly regressed onto their true values. We used the slope coefficient as an index of estimation accuracy. We also calculated the R-squared (coefficient of determination) between the estimated and true dietary proportions as an index of precision of the estimates.

### An Example Application to the Kenyan Grassland Food Web with Real Isotope Data

**Figure 1:**
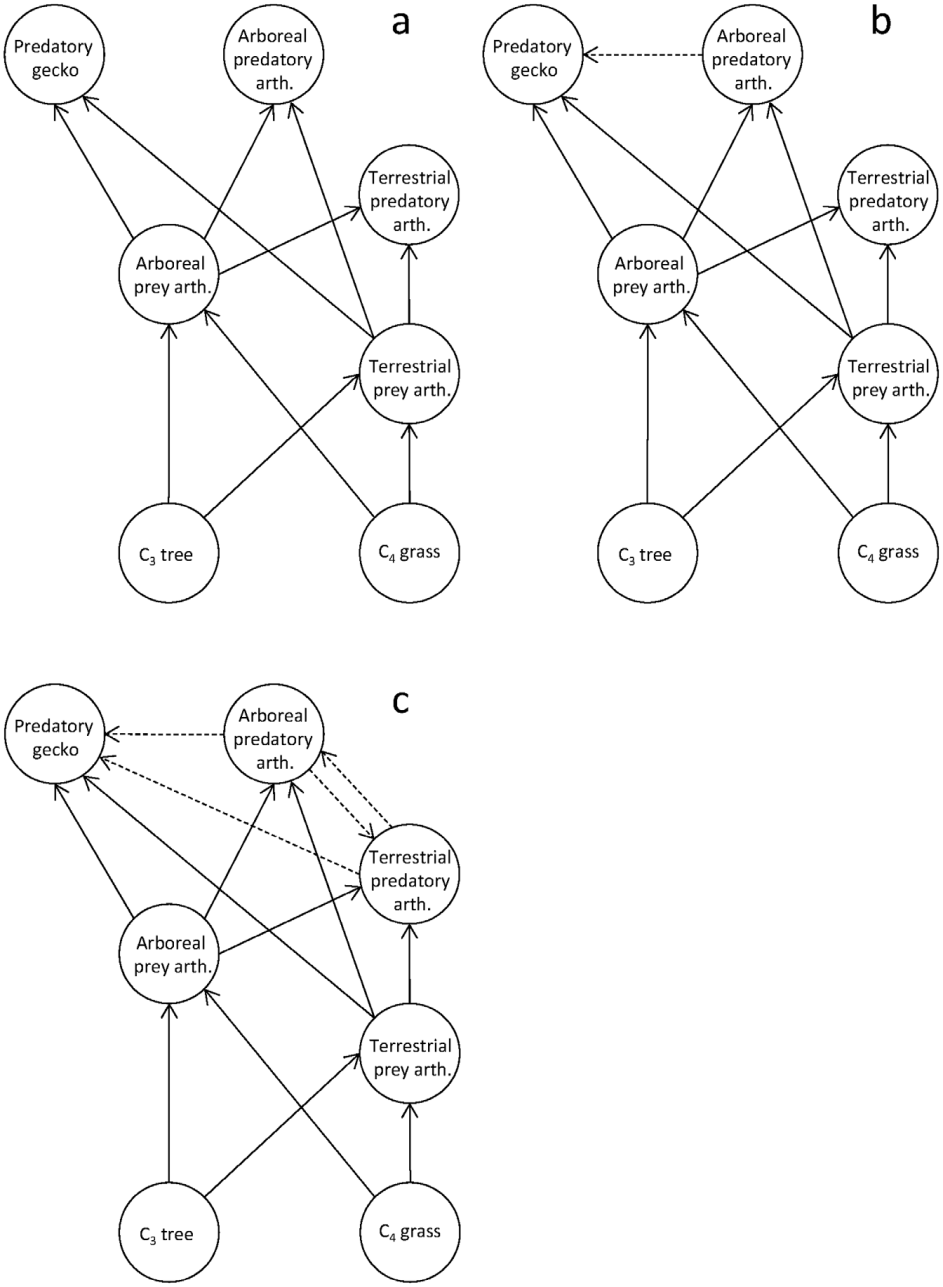
Topological web of the Kenyan wooded grassland food web analyzed in the present study: (a) the original topological web shown by Pringle & Fox–Dobbs [**11**], (b) topological web with the link from arboreal predatory arthropods to geckos added to the original topological web, and (c) topological web with all possible links between predatory species except geckos uneaten by predatory arthropods. We evaluated the alternative food-web topologies in terms of fits to data of isotope signatures using IsoWeb.

Pringle and Fox–Dobbs [11] used stable isotope analysis to quantify the structure of the food web of a Kenyan wooded grassland consisting of 7 species groups: C_3_ plants (trees and shrubs), C_4_ plants (grasses), arboreal prey arthropods (e.g., beetles), terrestrial prey arthropods (e.g., grasshoppers), arboreal predatory arthropods (e.g., spiders), terrestrial predatory arthropods (e.g., praying mantises), and arboreal geckos (*Lygodactylus keniensis*). The authors determined a topological web (Fig. 1a) by using inference from literature [22], [23], author's observation, and nitrogen isotope information (to infer predator-prey relationships). An isotope mixing model (IsoError [24]) was then applied individually to each consumer, in order to determine the dietary proportions of different resources used by each consumer. The mixing model used carbon isotope information only, because each consumer has only two resources in the assumed topological web (and the assumed topological web was based partly on nitrogen stable isotope information). To evaluate uncertainty in estimated dietary proportions due to potential variation in trophic enrichment factors, the authors used three alternative values in the carbon trophic enrichment factor (0.5, 1.5, or 2.5 ‰).

We applied IsoWeb to the original isotope data of the Kenyan wooded grassland food web, and compared IsoWeb’s estimates with the estimates by Pringle and Fox–Dobbs [11]. We applied IsoWeb to four scenarios. In the first scenario, we assumed the same topological web as of Pringle and Fox–Dobbs [11] (Fig. 1a), and used carbon isotope information only. In the second, we assumed the same topological web as of Pringle and Fox–Dobbs [11], and used both carbon and nitrogen isotope information. In the third, we added the link from arboreal predatory arthropods to geckos to the original topological web (Fig. 1b), and used both carbon and nitrogen isotope information. This additional link was based on the observation by Greer [22] that geckos utilized a wide variety of prey. In the forth, we assumed a topological web with all possible links between predatory species except that geckos would not be preyed upon by predatory arthropods (Fig. 1c), and used both carbon and nitrogen isotope information to estimate dietary proportions. This scenario simulated the case in which we did not have enough information from literature or field observations to determine the presence/absence of trophic links between predators. To compare different candidate topologies in the second, third, and fourth scenarios, we used the Bayes factor (BF). The BF was calculated for two combinations of candidate models (i.e., the second vs. third scenarios and the second and forth scenarios) according to the method described in Spiegelhalter et al. [25] in which alternative models (i.e., original topology and that in third or fourth scenarios) were combined into a single large model and relative probabilities of them were simultaneously evaluated as one of parameters in the course of parameter estimation of the large model. We assigned the same probabilities (i.e., 0.5) as priors for two competing models. We ran three MCMC chains in each estimation and checked the convergence of the relative probability parameters of candidate models based on trace plots.

## Results

### Sensitivity Analysis

**Figure 2:**
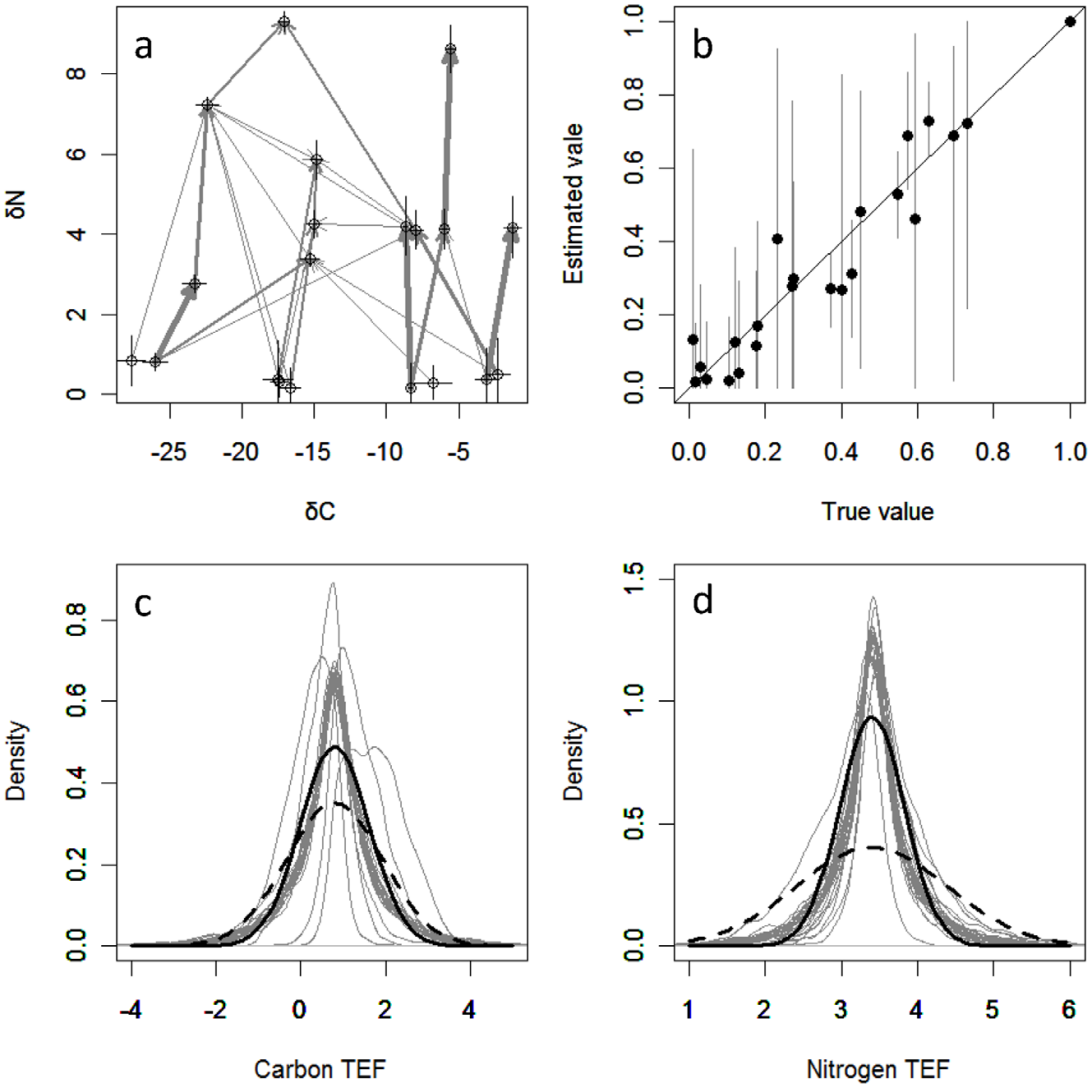
An example of the application of IsoWeb to a hypothetical food web. (a) δ^13^C–δ^15^N plot of the hypothetical web (parameter values: number of species  = 20; connectance  = 0.1; number of samples  = 10; residual errors  = 0.1 for carbon and nitrogen). Each point and grey arrow represent species and prey-predator interaction which are consisted in the hypothetical food web. (b) Comparison between true and estimated dietary proportions. The regression coefficient between true and estimated dietary contributions is 0.973, *p*<0.0001. Error bar represents 95% credible interval of the estimated dietary proportion and solid line represents symmetry line. (c, d) The posterior probability density of trophic enrichment factor (TEF) of carbon and nitrogen. IsoWeb takes into account between–link variation in TEFs. Thick dashed and solid line represent true and estimated probability density, and thin grey line represents posterior distribution of the estimated probability density for each link.

Fig. 2 illustrates an example of the application of IsoWeb to a hypothetical food web. Fig. 2a is the δ^13^C–δ^15^N plot of the hypothetical web (parameter values: *S* = 20; *C* = 0.1; *n* = 10; *σ_ij_* ^2^ = 0.1 for carbon and nitrogen). The estimated dietary contributions were generally close to the true values of the hypothetical web (Fig. 2b; the regression coefficient between true and estimated dietary contributions is 0.973, *p*<0.0001), showing that IsoWeb can make reasonably accurate estimates. However, the 95% credible interval of every estimated dietary contribution were quite large, reflecting that IsoWeb takes into account between–link variation in the trophic enrichment factors (Fig. 2c for carbon and 2d for nitrogen).

**Figure 3:**
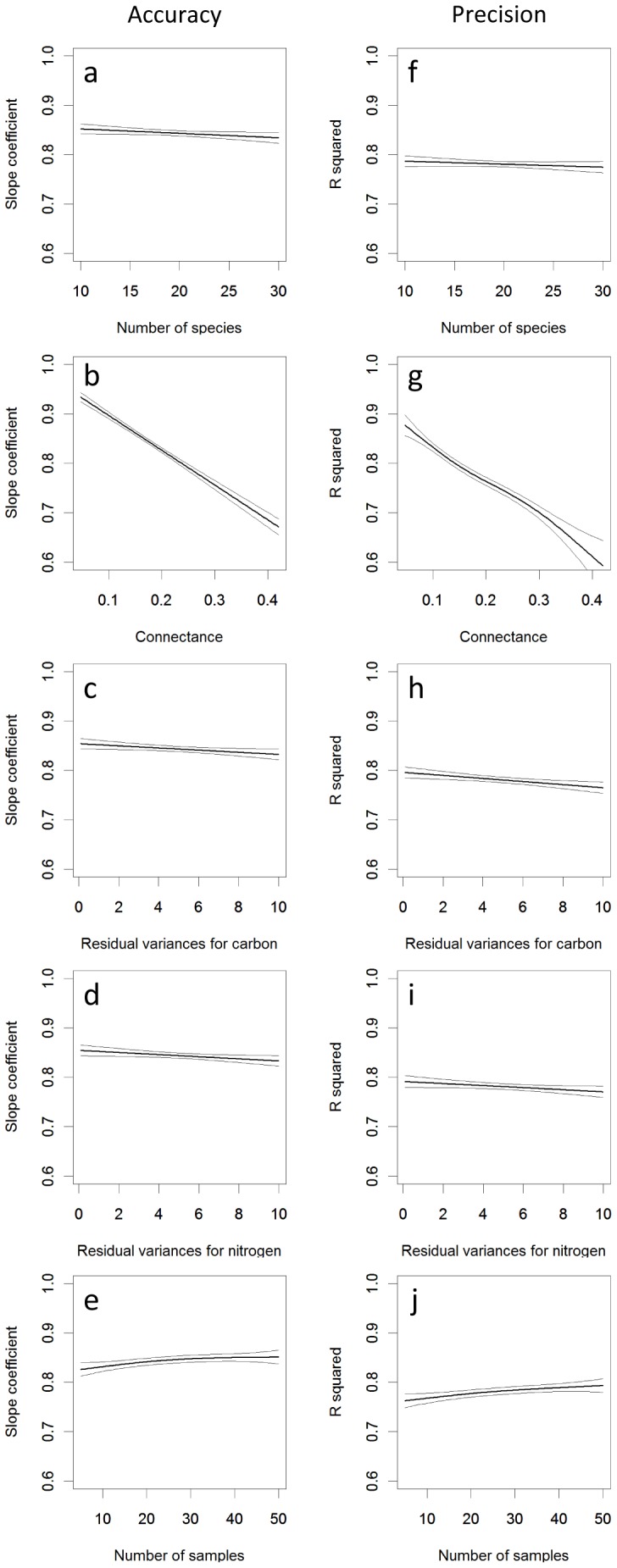
Sensitivity of IsoWeb to food-web parameters and sample size. Response of estimation accuracy and precision to (a, f) number of species, *S*; (b, g) connectance, *C*; (c, h) residual variances for carbon, *σ_ij_* ^2^; (d, i) residual variances for nitrogen, *σ_ij_* ^2^ and (e, j) number of samples, *n*. The slope coefficients and R-squared are indices of estimation accuracy and precision, respectively. Dashed line represents the doubled standard errors.

Sensitivity analysis against food-web parameters and sample size showed that IsoWeb estimates generally performed well for a wide variety of these parameters, although some food–web parameters could disturb the estimates (Fig. 3). Accuracy and precision showed similar sensitivity. Correspondence between true and estimated dietary proportions was most influenced by connectance, with the accuracy and precision of estimates decreasing with increasing connectance. Increasing food-web size and increasing the variance of carbon and nitrogen isotope ratios in each species also tended to lower the performance. Increasing the number of stable isotope samples from each species increased the performance.

**Figure 4:**
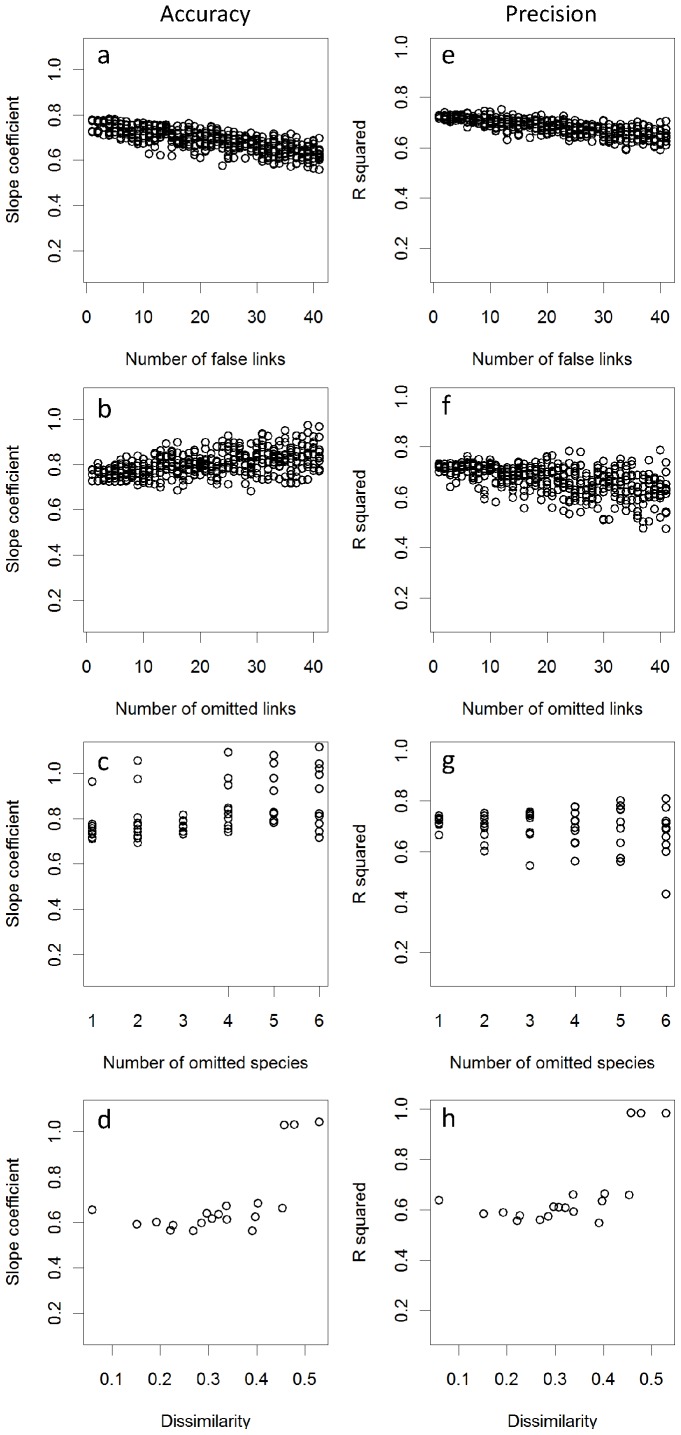
Sensitivity of IsoWeb to topological uncertainty in the Benguela food web. Responses of estimation accuracy and precision of IsoWeb to (a, e) number of false links; (b, f) number of omitted links; (c, g) number of omitted species and (d, h) dissimilarity (1 - Jaccard similarity) threshold for species aggregation. Slope coefficients and R-squared are indices of estimation accuracy and precision, respectively.

IsoWeb showed moderate sensitivity to topological uncertainty in the Benguela, the Coachella Valley, and the Small Reef food webs. Similar trends were found for these webs. Increasing the number of false links tended to decrease both accuracy and precision, whereas the increase in the number of omitted links slightly increased accuracy and decreased precision (Fig. 4, S3, S4). Increasing the number of omitted species did not affect either accuracy or precision. However, the slope coefficients could be higher than that for the original web (Fig. 4, S3, S4). Trophic aggregation slightly lowered both accuracy and precision. However, increasing the threshold dissimilarity for the aggregation did not strongly affect the accuracy and precision, except that accuracy and precision could be elevated at around a large dissimilarity.

### Application to the Kenyan Grassland Food Web

When only carbon isotope information was used (as in the original estimates [11]), IsoWeb yielded the estimates of dietary proportions similar to the original estimates (Table 1). The 95% credible intervals of IsoWeb estimates are generally four to ten times wider than the range of the original estimates provided in Pringle and Fox–Dobbs [11]. When both carbon and nitrogen isotope information was used for relaxed topological webs with additional potential trophic links among predators, the estimated dietary proportions of these additional links were low, suggesting that the original topological web in Pringle and Fox-Dobbs [11] was a reasonable assumption (Table 1). The 95% credible intervals based on both carbon and nitrogen information were generally smaller than those based only on carbon information.

**Table 1:** Estimated dietary proportion and its range for the Kenyan wooded grassland food web [11].

		Original estimates	IsoWeb estimates			
		Topological web				
		Original	Original	Original	With an intragulid predation link to gecko	With links among all predators
		Isotope information				
Predator	Prey	Carbon	Carbon	Carbon Nitrogen	Carbon Nitrogen	Carbon Nitrogen
Predatory gecko	Arboreal predatory arth.	–	–	–	0.11 [0.00, 0.85]	0.12 [0.00, 0.80]
	Terrestrial predatory arth.	–	–	–	–	0.05 [0.00, 0.53]
	Arboreal prey arth.	(0.89–0.99)	0.89 [0.06, 1.00]	0.90 [0.17, 1.00]	0.71 [0.02, 0.99]	0.59 [0.01, 0.95]
	Terrestrial prey arth.	(0.01–0.11)	0.11 [0.00, 0.94]	0.10 [0.00, 0.83]	0.08 [0.00, 0.80]	0.08 [0.00, 0.76]
Arboreal predatory arth.	Terrestrial predatory arth.	–	–	–	–	0.08 [0.00, 0.60]
	Arboreal prey arth.	(0.81–0.89)	0.90 [0.06, 1.00]	0.91 [0.16, 1.00]	0.90 [0.10, 1.00]	0.75 [0.02, 0.99]
	Terrestrial prey arth.	(0.11–0.19)	0.10 [0.00, 0.94]	0.09 [0.00, 0.84]	0.10 [0.00, 0.90]	0.10 [0.00, 0.86]
Terrestrial predatory arth.	Arboreal predatory arth.	–	–	–	–	0.15 [0.00, 0.75]
	Arboreal prey arth.	(0.31–0.52)	0.33 [0.00, 0.98]	0.32 [0.00, 0.95]	0.32 [0.00, 0.98]	0.22 [0.00, 0.88]
	Terrestrial prey arth.	(0.48–0.69)	0.67 [0.02, 1.00]	0.68 [0.05, 1.00]	0.68 [0.02, 1.00]	0.54 [0.01, 0.96]
Arboreal prey arth.	C_3_ tree	–	0.69 [0.16, 0.99]	0.69 [0.39, 0.99]	0.71 [0.31, 0.99]	0.71 [0.36, 0.99]
	C_4_ grass	–	0.31 [0.01, 0.84]	0.31 [0.01, 0.61]	0.29 [0.01, 0.69]	0.29 [0.01, 0.64]
Terrestrial prey arth.	C_3_ tree	–	0.17 [0.00, 0.61]	0.18 [0.00, 0.66]	0.19 [0.00, 0.77]	0.19 [0.00, 0.88]
	C_4_ grass	–	0.83 [0.39, 1.00]	0.82 [0.34, 1.00]	0.81 [0.23, 1.00]	0.81 [0.12, 1.00]
Bayes Factor		–	–	1.000	1.786	5.329

For IsoWeb results, median and 95% credible intervals (shown in the bracket) were shown. For description of the topological webs, see Fig. 1. Bayes factors were calculated between the data with original topology and those with the candidate topologies.

IsoWeb applied to different topological webs in the second, third, and fourth scenarios showed that IsoWeb can evaluate the plausibility of presence/absence of potential trophic links. BFs between two combinations of two candidate models were successfully calculated (Fig. S5). BF analysis found that the original topology (Fig. 1a) received some support (according to a rule of thumb of Jeffrery [26]) against the candidate topology with links among all predators (Fig. 1c), and little support over the topology with an intraguild predation link to geckos (Fig. 1b). Thus, the intraguild predation link from arboreal predatory arthropods to geckos may exist although the dietary contribution through this link can be small, whereas other links among predators may be unlikely.

## Discussion

We developed a Bayesian isotope mixing model, IsoWeb, to estimate diet proportions of all consumers in the whole food web. Sensitivity analysis using hypothetical food webs suggested that IsoWeb is applicable to a wide variety of natural food webs. In addition, sensitivity analysis using real topological webs suggested that IsoWeb can tolerate a certain level of topological errors and trophic aggregation. Moreover, we used published data [11] to demonstrate a successful application of IsoWeb to a real food web with real isotope data. Below we discuss the results of sensitivity analysis, issues dealing with variation in trophic enrichment factors, and future pointers for the use of IsoWeb in food-web quantification.

### Robustness to Food–web Parameters and Sample Size

The food-web parameter that influenced IsoWeb's performance most strongly was connectance, with increasing connectance lowering the performance. This is a problem inherent to stable-isotope mixing models applied to consumers utilizing potentially many resources, because increasing the number of links per species elevates uncertainty around estimated dietary proportions [27]. This problem could be overcome by increasing the number of tracers, such as sulfur and oxygen stable isotopes. IsoWeb allows straightforward implementation of such extension (see Supplement S1).

Sensitivity analysis demonstrated that IsoWeb requires only a relatively small number of samples (approx. 5 to 10 samples per node) to estimate consumer's dietary proportions with adequate accuracy and precision. Robustness of IsoWeb against small sample size is arguably because IsoWeb uses the information from all consumers and resources in a food web.

### Robustness to Topological Uncertainty

Increasing the number of false links decreased accuracy and precision, because IsoWeb does not estimate the dietary proportions of any links to be exact zero, and assuming a false link to a consumer lowers the estimated dietary proportions of existent links of this consumer. Increasing the number of omitted links increased accuracy, because random link omission tends to remove links with intermediate dietary contributions, and strengthen the relative effects of remaining links with extreme (i.e., nearly one or zero) dietary proportions on determining regression slopes in a way that increases the slopes. It is worth noting that because links with smaller dietary proportions are more likely to be overlooked in the field, our analysis assuming random link omission may overestimate the adverse effects of the uncertainty on the IsoWeb performance. Fairly robust performance of IsoWeb against species omission is probably because species omission necessarily involves removal of links connected to the omitted species. The observed increase of accuracy and precision around a high level of trophic aggregation is because aggregation leaves links whose dietary proportions can be easily estimated (e.g., a single link from a consumer).

### Incorporating Variation in Trophic Enrichment Factors

Trophic enrichment factors can vary depending on consumers' characteristics, such as diet composition and feeding rates [9], [28], [29]. Despite such substantial variability, most studies using isotope mixing models assume constant trophic enrichment factors, which could underestimate uncertainty or bring biases in estimated dietary proportions. To address these problems, IsoWeb allows trophic enrichment factors to vary among links. Specifically, IsoWeb assumes trophic enrichment factors as random variables drawn from a normal distribution, and estimates the variance of this normal distribution. This approach enables estimates that take into account uncertainty due to between–link variation in trophic enrichment factors, while avoiding biases resulting from assuming incorrect constant trophic enrichment factors. However, on the other hand, allowing for the uncertainty of trophic enrichment factors makes credible intervals of IsoWeb estimates inevitably wider than the estimates assuming constant trophic enrichment factors.

To take an example from the IsoWeb application to the Kenyan wooded grassland food web, the credible intervals of IsoWeb estimates are generally four to ten times wider than the range of the original estimates by Pringle and Fox–Dobbs [11]. This is largely because, while the original estimates assumed a constant trophic enrichment factor for different links, IsoWeb relaxes this assumption by allowing for between-link variation in the trophic enrichment factors. The wider credible intervals of IsoWeb estimates may suggest that the original analysis without between–link variation in trophic enrichment factors might underestimate uncertainty in the estimated dietary proportions.

The present analyses assigned 0.8 to the mean of the assumed normal distribution of carbon trophic enrichment factors, and 3.4 to that of nitrogen trophic enrichment factors. These values are based on the empirical distributions of carbon and nitrogen trophic enrichment factors from literature [9]. IsoWeb is easily customized to use different mean values where they are appropriate as well as customizing other parts of the model such as to use of a non-Gaussian probability distribution as priors for isotope ratio data (see Supporting Information 1).

### Future Prospects

Through our re–analysis of the Kenyan wooded grassland food web [11], we showed that IsoWeb can evaluate the plausibility of presence/absence of potential trophic links. This suggests that, given the stable isotope information of all consumers and resources in a food web, IsoWeb could compare the plausibility of different candidate topologies for this web on the basis of information criteria such as BF. In a field, there often are links that are difficult to observe, and IsoWeb may be useful to seek a likely topology in such a situation. Furthermore, topologies of real food webs can vary through space and time [30], [31]. With adequate isotope data, IsoWeb may be useful for determining which topologies are realized in different locations or time. We note that MixSIR [12] and SIAR [13] have a similar potential of evaluating the plausibility of possible links from candidate resources to a particular consumer. Yet IsoWeb may be superior in this respect, because IsoWeb can take advantage of utilizing a larger volume of information derived from all consumers and resources in a food web.

Overall, IsoWeb will provide a powerful method toward reconstructing quantitative food webs from stable isotope information. Estimating the dietary proportions of all consumers in a food web is a crucial step to quantify interaction strength for a whole food web, which provides necessary information to analyze the dynamics and stability of a food web [3], [32]. However, to conduct such analyses, determining an energy flow web is required. The present version of IsoWeb does not estimate an energy flow web. A promising future challenge is to develop a framework that can determine an energy flow web by integrating stable isotope and other information, such as the biomass and metabolic rate of each species.

## Supporting Information

Figure S1 **Comparison of estimation accuracy (a) and precision (b) between IsoWebs assuming normal distribution (Normal) and uniform distribution (Unifrom) for priors of trophic enrichment factors.** A total of 1500 hypothetical food webs and associated test data sets were generated, and the two models were applied to each of data sets and the performance of the estimation was assessed (see text for details). IsoWeb with uniform prior was significantly inferior in accuracy (p<0.0001, Wilcoxon test), whereas precision was not significantly different between the models with uniform and normal distributions (p = 0.232, Wilcoxon test). The result would be partly because uniform prior would be less informative than normal priors and accordingly posteriors of TEFs became more broad so that IsoWeb became prone to over and underestimate for lower and higher contribution rate, respectively. Because the bias was likely arise systematically (i.e., larger bias for higher or lower contribution rate and smaller bias for moderate contribution rate), precision measured by R-squared did not differed between the uniform and normal priors.
(TIF)

Figure S2 **Sensitivity of the IsoWeb with uniform prior to food-web parameters and sample size.** Response of estimation accuracy and precision to (a, f) number of species, *S*; (b, g) connectance, *C*; (c, h) residual variances for carbon, *σ_ij_* ^2^; (d, i) residual variances for nitrogen, *σ_ij_* ^2^ and (e, j) number of samples, *n*. The slope coefficients and R-squared are indices of estimation accuracy and precision, respectively. Dashed line represents the doubled standard errors.
(TIF)

Figure S3 **Sensitivity of IsoWeb to topological uncertainty in the Coachella Valley food web.** Responses of estimation accuracy and precision of IsoWeb to (a, e) number of false links; (b, f) number of omitted links; (c, g) number of omitted species and (d, h) dissimilarity (1 - Jaccard similarity) threshold for species aggregation. Slope coefficients and R-squared are indices of estimation accuracy and precision, respectively.
(TIF)

Figure S4 **Sensitivity of IsoWeb to topological uncertainty in the Small Reef food web.** Responses of estimation accuracy and precision of IsoWeb to (a, e) number of false links; (b, f) number of omitted links; (c, g) number of omitted species and (d, h) dissimilarity (1 - Jaccard similarity) threshold for species aggregation. Slope coefficients and R-squared are indices of estimation accuracy and precision, respectively.
(TIF)

Figure S5 **Trace plots of parameters that determine the probability of candidate models in comparisons between (a) original (the second scenario; model 1) vs. the third scenario topologies (model 2), and (b) original vs. the fourth scenario topologies (model 3).** Different colors in the plot represent MCMC chains with different random number series.
(TIF)

Supplement S1 **R script for IsoWeb.**
(R) Click here for additional data file.

Supplement S2 **Procedure for generating virtual stable isotope data.**
(DOC)

## References

[1] DeAngelis DL (1980) Energy-flow, nutrient cycling, and ecosystem resilience. Ecology 61: 764–771.

[2] McCann K, Hastings A, Huxel GR (1998) Weak trophic interactions and the balance of nature. Nature 395: 794–798.

[3] de Ruiter PC, Neutel A-M, Moore JC (1994) Modelling food webs and nutrient cycling in agro-ecosystems. Trends in Ecology & Evolution 9: 378–383. 21236897 10.1016/0169-5347(94)90059-0

[4] Vanni MJ (2002) Nutrient Cycling by Animals in Freshwater Ecosystems. Annual Review of Ecology and Systematics 33: 341–370.

[5] Paine RT (1980) Food webs - linkage, interaction strength and community infrastructure. Journal of Animal Ecology 49: 667–685.

[6] van Veen FJF, Morris RJ, Godfray HCJ (2006) Apparent competition, quantitative food webs, and the structure of phytophagous insect communities. Annual Review of Entomology 51: 187–208. 10.1146/annurev.ento.51.110104.151120 16332209

[7] Banasek-Richter C, Cattin MF, Bersier LF (2004) Sampling effects and the robustness of quantitative and qualitative food-web descriptors. Journal of Theoretical Biology 226: 23–32. 14637051 10.1016/s0022-5193(03)00305-9

[8] Newsome SD, Tinker MT, Monson DH, Oftedal OT, Ralls K, et al (2009) Using stable isotopes to investigate individual diet specialization in California sea otters (*Enhydra lutris nereis*). Ecology 90: 961–974. 19449691 10.1890/07-1812.1

[9] Post DM (2002) Using stable isotopes to estimate trophic position: Models, methods, and assumptions. Ecology 83: 703–718.

[10] West JB, Bowen GJ, Cerling TE, Ehleringer JR (2006) Stable isotopes as one of nature's ecological recorders. Trends in Ecology & Evolution 21: 408–414. 16753238 10.1016/j.tree.2006.04.002

[11] Pringle RM, Fox-Dobbs K (2008) Coupling of canopy and understory food webs by ground-dwelling predators. Ecology Letters 11: 1328–1337. 19046361 10.1111/j.1461-0248.2008.01252.x

[12] Moore JW, Semmens BX (2008) Incorporating uncertainty and prior information into stable isotope mixing models. Ecology Letters 11: 470–480. 18294213 10.1111/j.1461-0248.2008.01163.x

[13] Parnell AC, Inger R, Bearhop S, Jackson AL (2010) Source partitioning using stable isotopes: coping with too much variation. Plos One 5. 10.1371/journal.pone.0009672 PMC2837382 20300637

[14] Lunn DJ, Thomas A, Best N, Spiegelhalter D (2000) WinBUGS - A Bayesian modelling framework: concepts, structure, and extensibility. Statistics and Computing 10: 325–337.

[15] Gelman A (2006) Prior distributions for variance parameters in hierarchical models(Comment on an Article by Browne and Draper). Bayesian Analysis 1: 515–533.

[16] Gelman A, Carlin JB, Stern HS, Rubin DB (2003) Bayesian Data Analysis: Chapman & Hall/CRC.

[17] Williams RJ, Martinez ND (2000) Simple rules yield complex food webs. Nature 404: 180–183. 10724169 10.1038/35004572

[18] Yodzis P (1998) Local trophodynamics and the interaction of marine mammals and fisheries in the Benguela ecosystem. Journal of Animal Ecology 67: 635–658.

[19] Polis GA (1991) Complex trophic interactions in deserts - an empirical critique of food-web theory. American Naturalist 138: 123–155.

[20] Opitz S (1996) Trophic interactions in Caribbean coral reefs. ICLARM Technical Report 43

[21] Yodzis P, Winemiller KO (1999) In search of operational trophospecies in a tropical aquatic food web. Oikos 87: 327–340.

[22] Greer AE (1967) The ecology and behavior of two sympatric Lygodactylus geckos. Breviora 268: 1–16.

[23] Hardy LM, Crnkovic AC (2006) Diet of amphibians and reptiles from the Engare Ondare River Region of central Kenya, during the dry season. African Journal of Herpetology 55: 143–159.

[24] Phillips DL, Gregg JW (2001) Uncertainty in source partitioning using stable isotopes. Oecologia 127: 171–179. 24577646 10.1007/s004420000578

[25] Spiegelhalter DJ, Best NG, Carlin BR, van der Linde A (2002) Bayesian measures of model complexity and fit. Journal of the Royal Statistical Society Series B 64: 583–616.

[26] Jeffrery H (1961) Theory of Probability. Oxford: Oxford University.

[27] Phillips DL, Gregg JW (2003) Source partitioning using stable isotopes: coping with too many sources. Oecologia 136: 261–269. 12759813 10.1007/s00442-003-1218-3

[28] Vander Zanden MJ, Rasmussen JB (2001) Variation in delta N-15 and delta C-13 trophic fractionation: Implications for aquatic food web studies. Limnology and Oceanography 46: 2061–2066.

[29] McCutchan JH, Lewis WM, Kendall C, McGrath CC (2003) Variation in trophic shift for stable isotope ratios of carbon, nitrogen, and sulfur. Oikos 102: 378–390.

[30] Warren PH (1989) Spatial and temporal variation in the sturucture of a fresh-water food web. Oikos 55: 299–311.

[31] Winemiller KO (1990) Spatial and temporal variation in tropical fish trophic networks. Ecological Monographs 60: 331–367.

[32] Emmerson MC, Raffaelli D (2004) Predator-prey body size, interaction strength and the stability of a real food web. Journal of Animal Ecology 73: 399–409.

