# Supplementary figures and images for: IsoWeb: A Bayesian Isotope Mixing Model for Diet Analysis of the Whole Food Web
Source: PLoS One. 2012 Jul 27;7(7):e41057. doi: 10.1371/journal.pone.0041057 (PMC3407136; doi:10.1371/journal.pone.0041057)

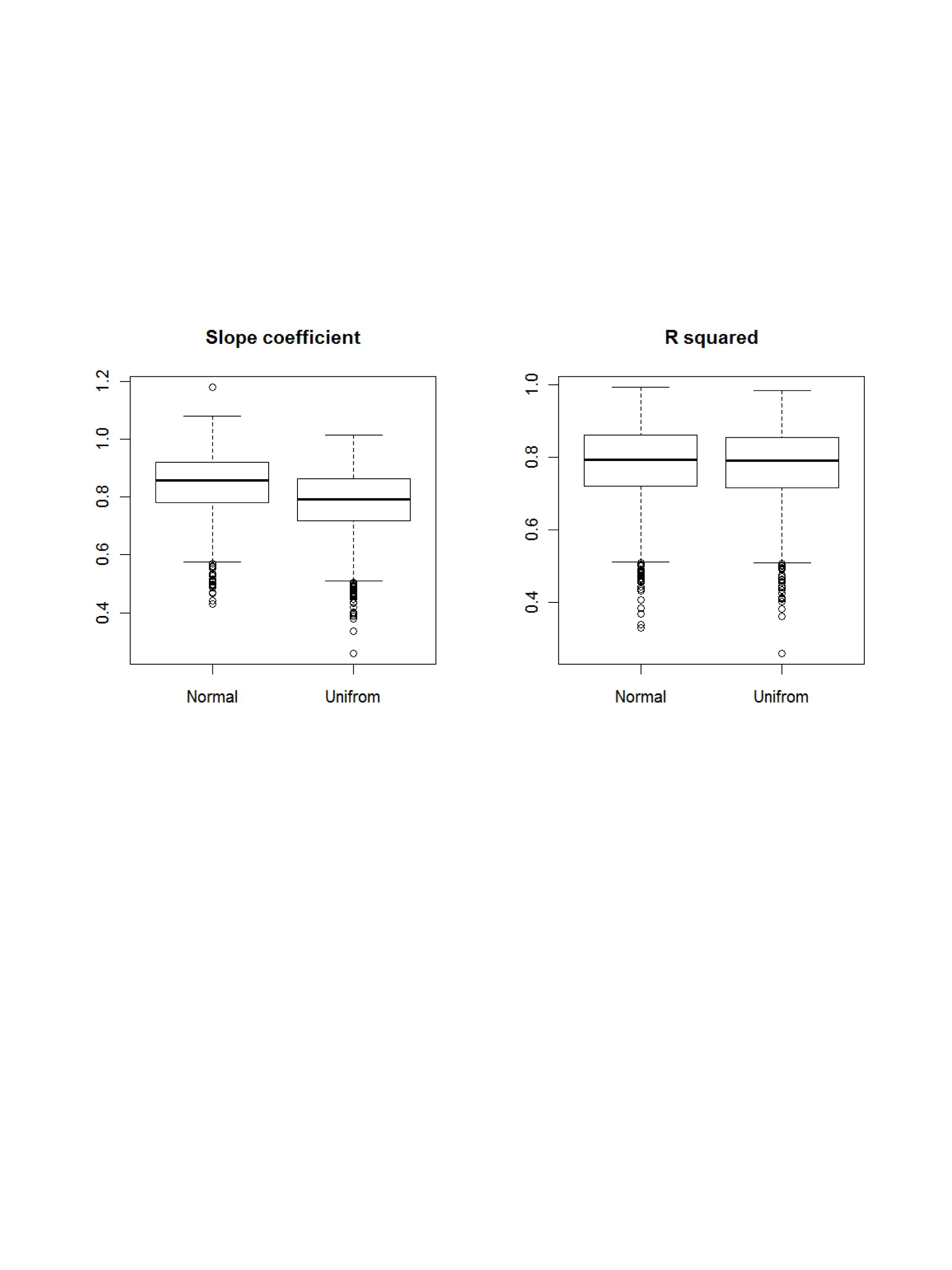

Supplement: Figure S1 — Comparison of estimation accuracy (a) and precision (b) between IsoWebs assuming normal distribution (Normal) and uniform distribution (Unifrom) for priors of trophic enrichment factors. A total of 1500 hypothetical food webs and associated test data sets were generated, and the two models were applied to each of data sets and the performance of the estimation was assessed (see text for details). IsoWeb with uniform prior was significantly inferior in accuracy (p<0.0001, Wilcoxon test), whereas precision was not significantly different between the models with uniform and normal distributions (p = 0.232, Wilcoxon test). The result would be partly because uniform prior would be less informative than normal priors and accordingly posteriors of TEFs became more broad so that IsoWeb became prone to over and underestimate for lower and higher contribution rate, respectively. Because the bias was likely arise systematically (i.e., larger bias for higher or lower contribution rate and smaller bias for moderate contribution rate), precision measured by R-squared did not differed between the uniform and normal priors. (TIF) [file pone.0041057.s001.tif]

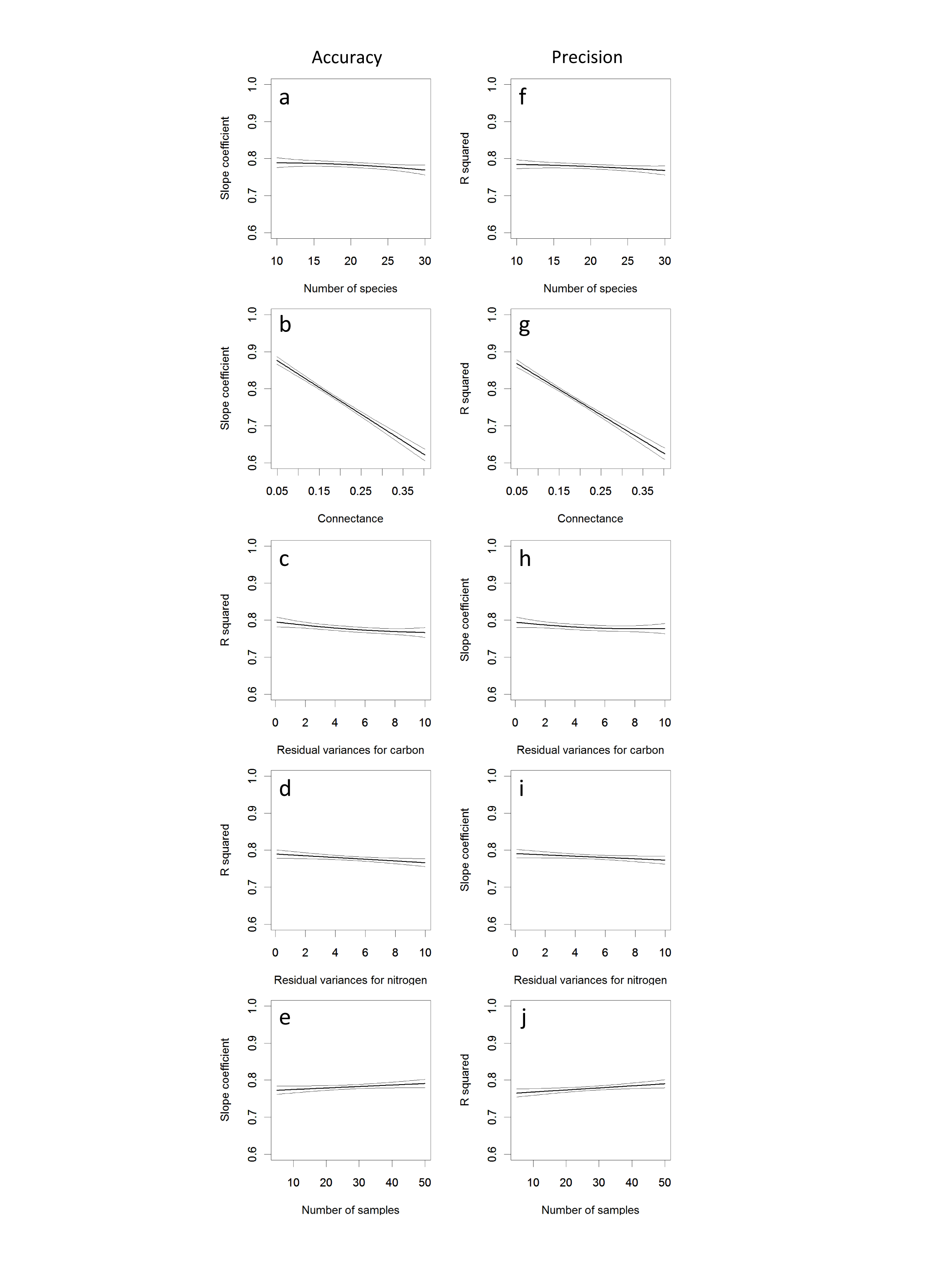

Supplement: Figure S2 — Sensitivity of the IsoWeb with uniform prior to food-web parameters and sample size. Response of estimation accuracy and precision to (a, f) number of species, S; (b, g) connectance, C; (c, h) residual variances for carbon, σij 2; (d, i) residual variances for nitrogen, σij 2 and (e, j) number of samples, n. The slope coefficients and R-squared are indices of estimation accuracy and precision, respectively. Dashed line represents the doubled standard errors. (TIF) [file pone.0041057.s002.tif]

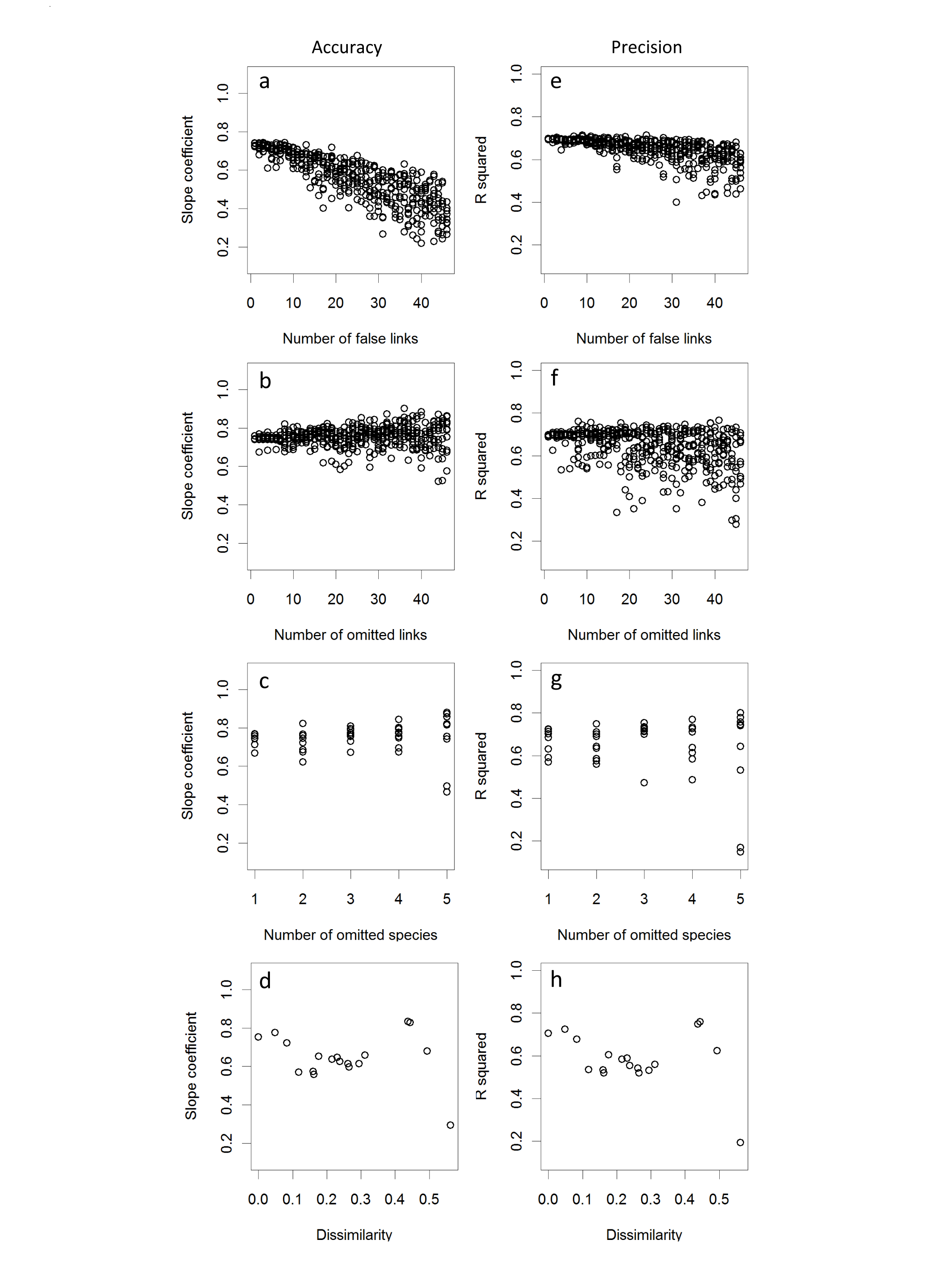

Supplement: Figure S3 — Sensitivity of IsoWeb to topological uncertainty in the Coachella Valley food web. Responses of estimation accuracy and precision of IsoWeb to (a, e) number of false links; (b, f) number of omitted links; (c, g) number of omitted species and (d, h) dissimilarity (1 - Jaccard similarity) threshold for species aggregation. Slope coefficients and R-squared are indices of estimation accuracy and precision, respectively. (TIF) [file pone.0041057.s003.tif]

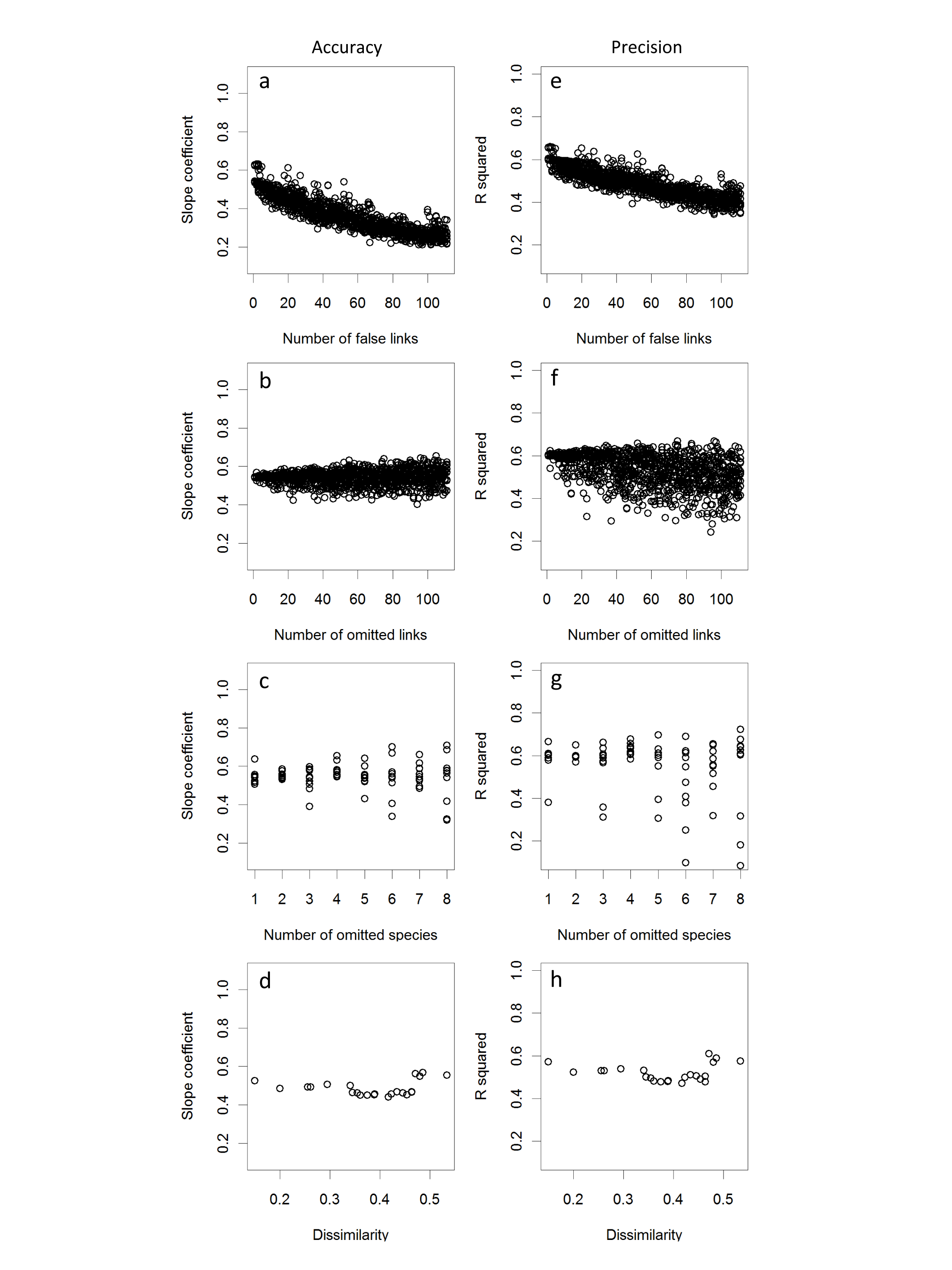

Supplement: Figure S4 — Sensitivity of IsoWeb to topological uncertainty in the Small Reef food web. Responses of estimation accuracy and precision of IsoWeb to (a, e) number of false links; (b, f) number of omitted links; (c, g) number of omitted species and (d, h) dissimilarity (1 - Jaccard similarity) threshold for species aggregation. Slope coefficients and R-squared are indices of estimation accuracy and precision, respectively. (TIF) [file pone.0041057.s004.tif]

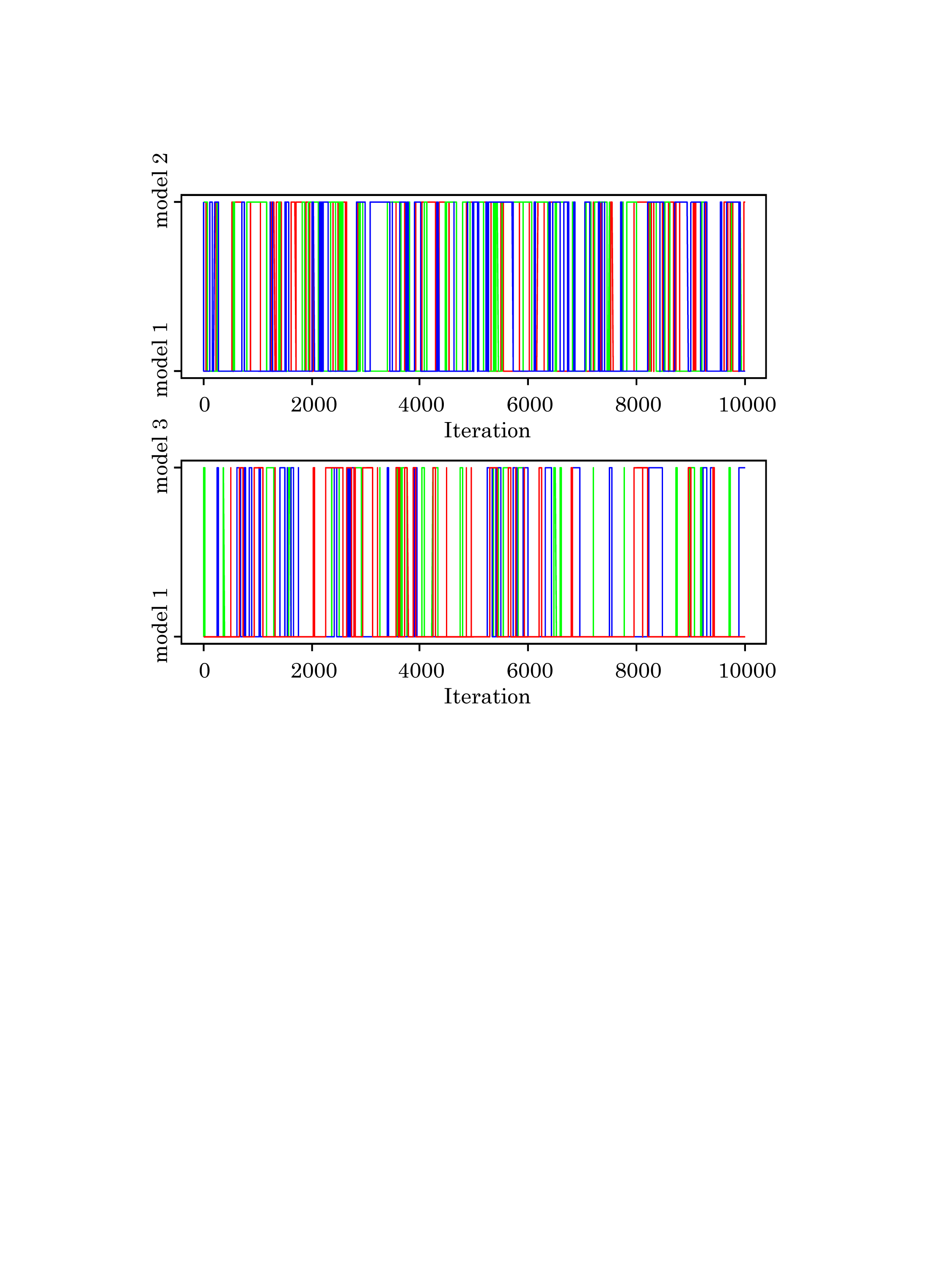

Supplement: Figure S5 — Trace plots of parameters that determine the probability of candidate models in comparisons between (a) original (the second scenario; model 1) vs. the third scenario topologies (model 2), and (b) original vs. the fourth scenario topologies (model 3). Different colors in the plot represent MCMC chains with different random number series. (TIF) [file pone.0041057.s005.tif]
