# Supplementary material for: IsoWeb: A Bayesian Isotope Mixing Model for Diet Analysis of the Whole Food Web
Source: PLoS One. 2012 Jul 27;7(7):e41057. doi: 10.1371/journal.pone.0041057 (PMC3407136; doi:10.1371/journal.pone.0041057)
Supplement: Supplement S2 — Procedure for generating virtual stable isotope data. (DOC) [file pone.0041057.s007.doc]

The procedure for generating test data sets was outlined. Without loss of generality, we assumed that concentrations of all elements considered are identical (i.e.,
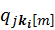
= 1).

1. Generate a topological web using the niche model with arbitrary *S* and *C*.

2. Generate the dietary proportions of different resources for each consumer randomly. These values are considered as true values of
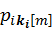
. We assumed that they follow a Dirichlet distribution in which all shape parameters were equally 0.5.

3. Assign isotope ratios randomly to basal resource species (i.e., those consume no other species). We considered two elements (i.e., carbon and nitrogen), and their isotope ratios in basal resource species were drawn from a uniform distribution of *U*[–30, 0] for carbon and *U*[0, 5] for nitrogen.

4. Determine a trophic enrichment factor of element *j* for each link from resource ***k****_i_*[*m*] to consumer *i*. These values are considered as true values of
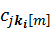
. We assumed that trophic enrichment factors follow a normal distribution of *N*(0.8, 1.3) for carbon and *N*(3.4, 0.98) for nitrogen (Post 2002).

5. Calculate the isotope ratios of each consumer
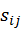
 as
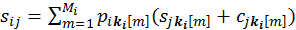
. These values are considered as true values.

6. Assign arbitrary constant value to the residual variances of consumer *i* on element *j* as true value of *σ_ij_*^2^. Without loss of generality, we assumed that the residual errors of all consumers are the same.

7. Generate an arbitrary sample size *n* of isotope ratio data for each species with the values of isotope ratios drawn randomly from *N* (*s_ij_*, *σ_ij_*^2^).

To generate realistic test data sets, we choose the following ranges for *S*, *C*, *n*, and *σ_ij_*^2^: *S* = 10 – 30, *C* = 0.05 – 3, *n* = 5 – 50, and *σ_ij_*^2^ = 0.1 – 10.0 (for carbon and nitrogen). Assuming parameter values randomly drawn from these ranges, a total of 1500 hypothetical food webs and associated test data sets were generated.
